# Genistein Improves Skin Flap Viability in Rats: A Preliminary In Vivo and In Vitro Investigation

**DOI:** 10.3390/molecules23071637

**Published:** 2018-07-04

**Authors:** Lenka Fáber, Ivan Kováč, Petra Mitrengová, Martin Novotný, Lenka Varinská, Tomáš Vasilenko, Martin Kello, Matúš Čoma, Tomáš Kuruc, Klaudia Petrová, Ivana Miláčková, Anika Kuczmannová, Vlasta Peržeľová, Štefánia Mižáková, Erik Dosedla, František Sabol, Ján Luczy, Milan Nagy, Jaroslav Majerník, Martin Koščo, Pavel Mučaji, Peter Gál

**Affiliations:** 1 Department of Pharmacology, Faculty of Medicine, Pavol Jozef Šafárik University, 040 11 Košice, Slovakia; lenka.faber@yahoo.com (L.F.); lenkavarinska@yahoo.com (L.V.); kellomartin@yahoo.com (M.K.); coma.matus@gmail.com (M.Č.); tomaskuruc@centrum.sk (T.K.); claudy.petrova@gmail.com (K.P.); 2 Department of Biomedical Research, East-Slovak Institute of Cardiovascular Diseases, 040 11 Košice, Slovakia; ivankovac.kovi@gmail.com (I.K.); martin.novotny.dr@gmail.com (M.N.); tomasvasilenko@gmail.com (T.V.); vlasta.perzelova@gmail.com (V.P.); 3 2nd Clinic of Surgery, Louis Pasteur University Hospital and Pavol Jozef Šafárik University, 041 90 Košice, Slovakia; 4 Department of Pharmacognosy and Botany, Comenius University, Faculty of Pharmacy, 832 32 Bratislava, Slovakia; peta.mitrengova@fpharm.uniba.sk (P.M.); imilackova@gmail.com (I.M.); kuczmannova@fpharm.uniba.sk (A.K.); nagy@fpharm.uniba.sk (M.N.); 5 Clinic of Infectology and Travel Medicine, Louis Pasteur University Hospital and Pavol Jozef Šafárik University, 041 90 Košice, Slovakia; 6 Clinic of Surgery, 1st Private Hospital Košice-Šaca and Pavol Jozef Šafárik University, 040 15 Košice, Slovakia; 7 Clinic of Heart Surgery, East-Slovak Institute of Cardiovascular Diseases and Pavol Jozef Šafárik University, 040 11 Košice, Slovakia; smizakova@vusch.sk (Š.M.); fsabol@vusch.sk (F.S.); jluczy@vusch.sk (J.L.); 8 Clinic of Obstetrics and Gynecology, 1st Private Hospital Košice-Šaca and Pavol Jozef Šafárik University, 040 15 Košice, Slovakia; edosedla@gmail.com; 9 Department of Medical Informatics, Faculty of Medicine, Pavol Jozef Šafárik University, 040 11 Košice, Slovakia; jaroslav.majernik@upjs.sk; 10 Clinic of Angiology, East-Slovak Institute of Cardiovascular Diseases and Pavol Jozef Šafárik University, 04011 Košice, Slovakia; mkosco@vusch.sk

**Keywords:** wound healing, skin repair, ischemia, estrogen, flap surgery, endothelial cell, SOD, CAT, Bcl-2

## Abstract

Selective estrogen receptor modulators (SERMs) have been developed to achieve beneficial effects of estrogens while minimizing their side effects. In this context, we decided to evaluate the protective effect of genistein, a natural SERM, on skin flap viability in rats and in a series of in vitro experiments on endothelial cells (migration, proliferation, antioxidant properties, and gene expression profiling following genistein treatment). Our results showed that administration of genistein increased skin flap viability, but importantly, the difference is only significant when treatment is started 3 days prior the flap surgery. Based on our in vitro experiments, it may be hypothesized that the underlying mechanism may rather by mediated by increasing SOD activity and Bcl-2 expression. The gene expression profiling further revealed 9 up-regulated genes (angiogenesis/inflammation promoting: CTGF, CXCL5, IL-6, ITGB3, MMP-14, and VEGF-A; angiogenesis inhibiting: COL18A1, TIMP-2, and TIMP-3). In conclusion, we observed a protective effect of genistein on skin flap viability which could be potentially applied in plastic surgery to women undergoing a reconstructive and/or plastic intervention. Nevertheless, further research is needed to explain the exact underlying mechanism and to find the optimal treatment protocol.

## 1. Introduction

Due to the increased average age of the population, almost a third of a woman’s life is characterized by estrogen deprivation related to numerous age-related processes, including poor wound healing and decreased skin flap viability [1,2]. Reconstructive surgical interventions frequently use flap techniques to help restore the integrity of a defected organ. Tissue ischemia resulting in flap necrosis and poor wound healing are the most frequently occurring and serious complications responsible for the failure of a successful surgery [3]. Accordingly, additional surgical interventions are inevitable, which increase both patients’ stress and hospitalization expenses [4]. It has been documented that therapeutic administration of pentoxifylline [5], nifedipine [6], sildenafil citrate [7], growth factors [4], hyperbaric oxygen therapy [8], phytotherapy [9], and/or low-level laser therapy [10] are able to enhance blood perfusion, prevent necrosis, and improve skin repair. However, some approaches have remained controversial, and/or demonstrated low efficiency.

It has also been well demonstrated that mice pretreated with estradiol developed an ischemia resistant environment resulting in the reperfusion of a protected vascular network which led to an increase of skin flap viability [2]. However, several side-effects of estrogen replacement therapy (ERT) preclude its common use in clinical practice. To minimize the detrimental side effects of ERT, selective estrogen receptor modulators (SERMs) have been developed [11]. The phytoestrogen genistein has also been considered as a natural SERM [12] and might play a preventive role in impaired wound healing, omitting the harmful estrogenic side effects on breast and/or uterine tissues [13]. In addition, genistein also exerts several antioxidant properties via regulating antioxidant enzyme activities such as SODs, heme oxygenase-1 (HO-1) and GPx [14], thus neutralizing reactive oxygen species (ROS). Moreover, dietary genistein supplementation accelerated wound healing by regulation of the antioxidant defense system and pro-inflammatory cytokines [15].

In this work, we used a rat model of skin flap ischemia to investigate whether genistein, a natural SERM, can prevent necrosis after blood flow impairment. We answered the question whether necrosis appeared in the distal and/or medial portion of the skin flap within 1 week following surgery in ovariectomized rats; it was reduced or even prevented in genistein-treated animals. We then evaluated the genistein-induced mechanisms responsible for this protection on the in vitro level, such as angiogenesis-related gene expression profiling, anti-oxidant properties, migration/proliferation, and vessel sprouting in endothelial cells.

## 2. Results

### 2.1. Macroscopic Analysis of Flap Survival

**Figure 1:**
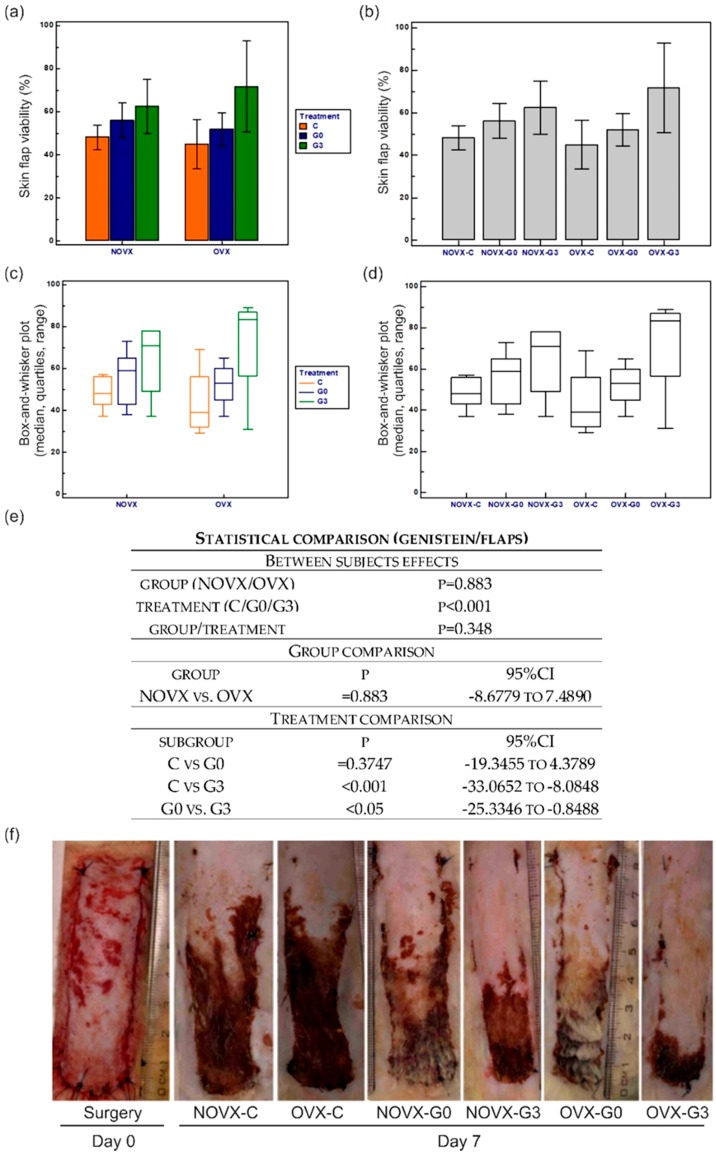
Skin flap viability expressed as a percentage of the original flap area generated on the day of surgery. (**a**) Between group comparison, values of individual groups with error bars (95% CI for the mean). (**b**) Treatment comparison, values of individual groups with error bars (95% CI for the mean). (**c**) Box-and-whisker plot of group comparison. (**d**) Box and whisker plot of treatment comparison. (**e**) Statistical analysis. (**f**) Macroscopic photographs of skin flaps. For group abbreviation explanation please see Table 1.

The percentage and statistical analysis of flap viabilities among the groups can be seen in Figure 1. Our study clearly demonstrated that genistein treatment is able to significantly increase skin flap viability in female rats. The highest flap survival rate was seen in OVX-G3 animals, while the lowest in the OVX-C group. Although the two-way analysis of variance (ANOVA) revealed poor differences between control and ovariectomised animals, the treatment comparison clearly indicates that genistein-mediated skin ischemia protective effect may only be found effective when treatment starts three days prior to the flap surgery and not at the day of flap surgery.

### 2.2. Flap Histology

**Figure 2:**
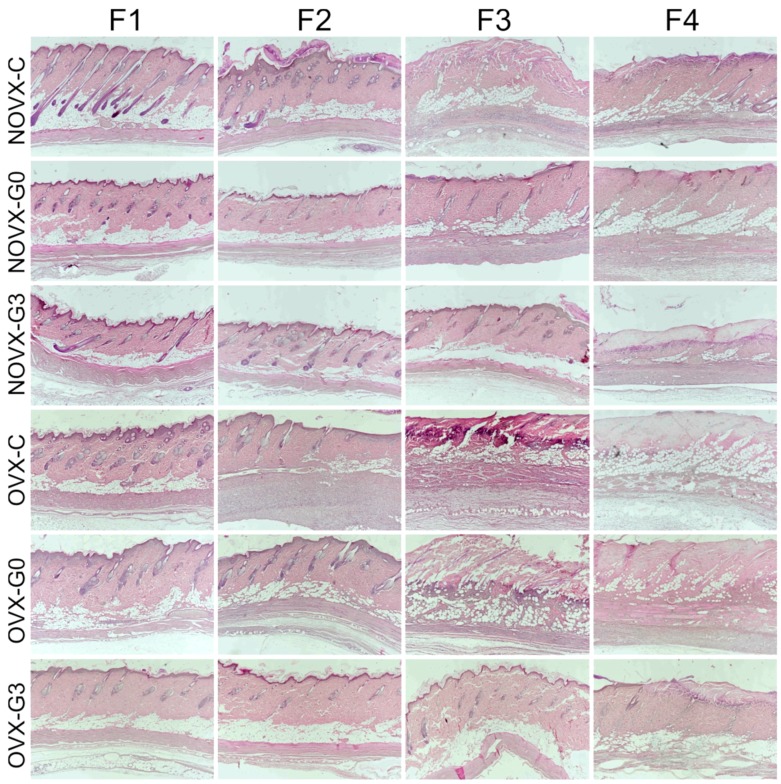
Representative histological figures of skin flap parts (from proximal F1 to distal F4) (magnification 40×, staining H + E; for group abbreviation explanation please see Table 1).

Representative histological pictures of selected skin flaps can be seen in Figure 2. Histological analysis was performed to compare the macroscopically observed ischemic lesions with the skin structure. Ovariectomy increased both density and size of pilosebaceous units, and increased skin thickness and occurrence of fat tissue. Necrosis was detected by microscopic examination at the same time as by macroscopic examination.

**Figure 3:**
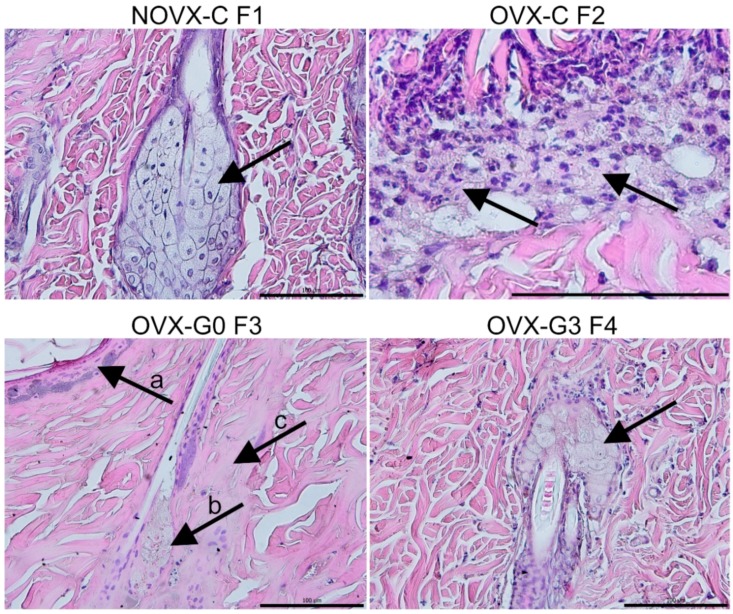
Representative details of cell/tissue changes following skin ischemia in selected groups/flap parts: NOVX-C F1 normal tissue, arrow point on undamaged sebaceous gland; OVX-C F2 demarcation line (rich on polymorphonuclear leucocytes) separating necrotic and vital tissue in the distal end of F2; OVX-G0 F3 damaged tissue with necrosis of epithelium, ghost cells in sebaceous gland and necrotic collagen fibers; OVX-G3 F4 (magnification: F1, F3, F4 = 400× and F2 = 1000×; scale 100 μm; staining H + E; for group abbreviation explanation please see Table 1).

The proximal part (Figure 2, F1) of all flaps demonstrated normal blood flow with no signs of tissue ischemia (for detail on hair follicle and sebaceous gland please see Figure 3, F1). The two medial flap parts (Figure 2, F2 and F3) exhibited divergent progression of degeneration toward necrosis. Here, differently located areas rich in polymorphonuclear leukocytes forming the demarcation line separating vital and ischemic/necrotic tissue were found. Whereas in ovariectomized untreated groups the demarcation lines and leukocyte infiltrations were seen in the second part (Figure 2, F2) of the flap, the genistein treatment extended flap survival so the demarcation line was seen in the third part (F3) of the flap similarly as seen in the non-ovariectomised control group. Furthermore, the demarcation lines in animals in which treatment started 3 days prior to the flap surgery were located between the third and fourth part (F3/F4) of their skin flaps. Tissue degeneration toward necrosis was prominent in medial parts (F3), mainly in hair follicle cells, sebaceous glands, and epidermal cells, as indicated by the presence of so-called “ghost cells” (for detail on epidermis (arrow-a), hair follicle/sebaceous gland (arrow-b) and collagen structure (arrow-c) please see Figure 3, F3). A similar picture was seen in the distal parts of skin flaps removed from genistein-treated rats (for detail please see Figure 3, F4). However, the distal portions (Figure 2, F4) of flaps removed from untreated rats as well as all distal ends of skin flaps from all rats were completely damaged, with the loss of tissue integrity throughout all skin layers (not shown).

### 2.3. Proliferation and Migration of Endothelial Cells

In order to evaluate cell viability of HUVECs cultured with different concentrations of genistein (1–100,000 nM) in the presence or absence of human recombinant VEGF (VEGF, 25 ng/mL) for 48 h the MTS-assay was carried out. Genistein alone or in combination with VEGF at 10,000 and 100,000 nM exerted cytotoxic effects resulted in significant inhibition of endothelial cell proliferation. However, genistein did not significantly affect cell viability at concentrations 1, 10, 100, and 1000 nM (data not shown). Therefore, for subsequent experiments only non-toxic concentrations in the range of 1–1000 nM were used.

**Figure 4:**
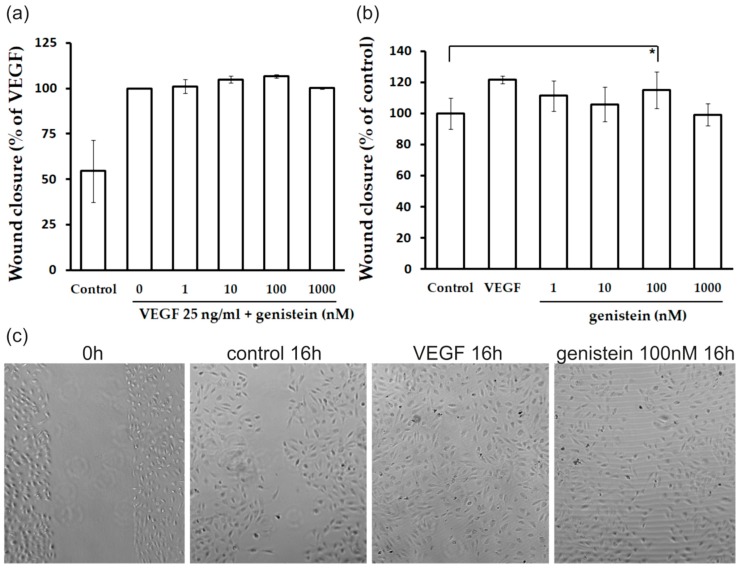
Influence of genistein on endothelial cell migration. (**a**) Confluent monolayer of HUVECs was wounded at 0 h. Subsequently, the cells were cultured with genistein for 16 h at different concentrations (1–1000 nM) in the presence of 25 ng/mL human recombinant VEGF. Values are mean ± SD from 2 cultures in 4 independent experiments. (**b**) Confluent monolayer of HUVECs was wounded at 0 h. Subsequently, the cells were cultured with genistein for 16 h at different concentrations (1–1000 nM) in the absence of VEGF. Values are mean ± SD from 2 cultures in 4 independent experiments (* *p* < 0.05 versus control). (**c**) Representative pictures of wound healing assay (magnification 100×).

The “wound healing” model of migration was used to evaluate the effect of genistein on endothelial cell migration. Confluent scrape-wounded HUVECs monolayers were incubated for 16 h with genistein (1–1000 nM) in the presence or absence of VEGF (25 ng/mL). Subsequently, the degree of closure of the “wound” was assessed. Genistein treatment alone at the concentration of 100 nM resulted in 15% stimulation of migration compared to untreated control (and 6% stimulation compared to VEGF control in the presence of VEGF) without any sights of cytotoxicity (Figure 4).

In order to work with a cell growth-stimulating concentration, we selected the concentration of 100 nM of genistein for gene profiling of HUVECs.

### 2.4. Gene Expression Profile of Endothelial Cells

To identify target genes involved in angiogenesis and vascular homeostasis regulated by genistein, we profiled HUVECs, using a commercial angiogenesis RT2 Profiler PCR Array. Among the 84 genes analyzed, 9 showed a significant up-regulation (≥ two fold difference; *p* value ≤ 0.05) in HUVECs treated with genistein (100 nM) in the presence of VEGF compared to VEGF control (Table 2). Of note, no genes were found to be down-regulated following genistein treatment in the presence of VEGF compared to VEGF control.

On the other hand, the expression of these genes was not deregulated in HUVECs after treatment with genistein in the absence of VEGF. Under this treatment, only two genes were down-regulated (CXCL10 and SERPINF1) compared to control (Table 3).

### 2.5. Bcl-2 Quantification after Genistein Treatment

**Figure 5:**
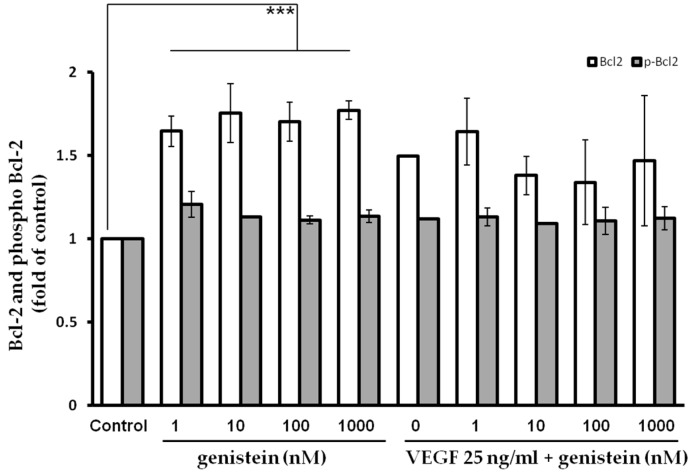
Analysis of mitochondrial apoptotic pathway associated protein Bcl-2. Relative levels and phosphorylation of Bcl-2 after genistein treatment. Significance: *** *p* < 0.001 vs. untreated cells (control).

To analyze changes on protein levels and activity following VEGF (25 ng/mL) and genistein (1, 10, 100, and 1000 nM) treatment and in mutual combination, the flow cytometric analyses of anti-apoptotic (Bcl-2) protein was performed (Figure 5). As the results showed, phosphorylation (responsible for activity inhibition) of Bcl-2 protein remained at low levels after VEGF, genistein, or combination treatment compared to the untreated control group and no significant change was observed. On the other hand, genistein induced significant increase in Bcl-2 levels compared to control. After mutual combination treatment (genistein with VEGF), a significant effect was not determined compared to VEGF alone.

### 2.6. CAT and SOD Activity in Endothelial Cells

**Figure 6:**
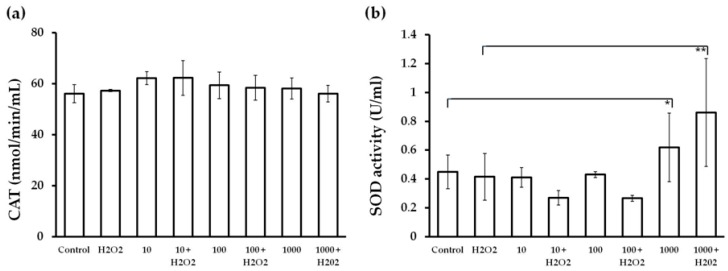
CAT (**a**)/SOD (**b**) activities after pre-treatment of HMVEC-D cells with different concentration (10, 100, and 1000 nM) of genistein (* *p* < 0.05; ** *p* < 0.01).

The effect of genistein (pre-treatment) on CAT activity can be seen in Figure 6a. None of the used concentration of genistein significantly altered the activity of CAT neither in HMECs (Figure 6a) nor in HUVECs (data not shown).

On the other hand, as it can be seen in Figure 6b, only pre-treatment with genistein increased the activity of SOD at the concentration of 1000 nM in HMVECs. Furthermore, in combination with hydrogen peroxide, the activity of SOD was even higher (Figure 6b). Furthermore, experiments conducted on HUVECs revealed similar tendency of SOD activity following genistein treatment (data not shown).

### 2.7. Genistein and ROS

**Figure 7:**
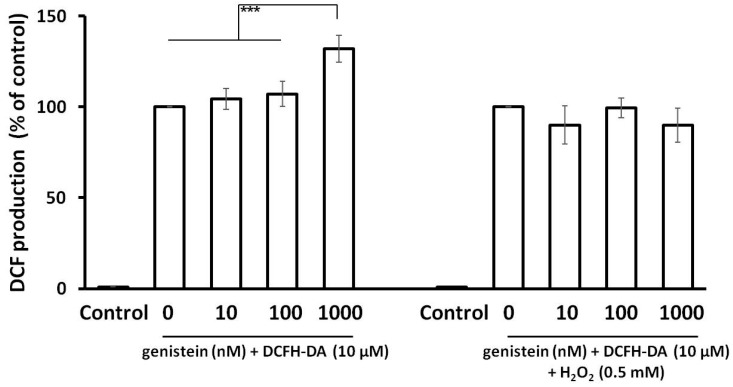
The effect of genistein on the generation of ROS in endothelial cell line HMVECs. The cells were seeded and incubated for 24 h. The production of DCF fluorescence depended on intracellular ROS content. The fluorescence was measured (λ (excitation/emission) = 480/530) after 2 h incubation with genistein (10, 100, and 1000 nM) and 1 h incubation with hydrogen peroxide (0.5 mM) or vehicle, respectively (*** *p* < 0.001).

The effect of genistein (pre-treatment) on ROS production can be seen in Figure 7. Genistein at the tested concentrations acted neither as an antioxidant nor as a prooxidant, except for the significant prooxidative effect of the highest tested genistein concentration in the absence of hydrogen peroxide.

## 3. Discussion

In the present study, we showed for the first time, to our knowledge, that genistein is able to significantly increase skin flap viability in female rats. Similarly as seen in two previous estrogen papers [2,16], the maximal protection was maintained with a 3-day pretreatment and not when initiated at the day of flap surgery. Therefore, it may be speculated that functional and/or structural changes in skin and/or vasculature seemed to be a prerequisite for the protective effect of genistein. The differences between genistein- and estradiol-mediated protective effects may be related to the fact that genistein also binds ERs, but its effect is weaker than the effect of estradiol. Moreover, genistein predominantly binds ER-β [17,18] and the estrogen-mediated skin tissue ischemia protective pathway includes rather ER-α than ER-β [2]. On the other hand, knock-out of ER-β led to significantly higher heart damage following ischemia-reperfusion injury as compared to wild-type animals. Treatment with both estradiol and a selective ER-β agonist led to increased S-nitrozylation of proteins and to cardioprotection [19]. Nevertheless, neuroprotective effects were mediated rather via ER-α [20,21], but the estrogen positive effects on skin repair are mediated rather via ER-β [22,23].

Previously, it has been demonstrated that the effect of estrogen against skin ischemia involves preservation of skin viability and enhancement of the anti-apoptotic Bcl-2 expression in vivo [2]. Looking in detail on selected skin cells, estradiol enhances Bcl-2 synthesis in cultured keratinocytes [24] and endothelial cells [25]. Our present in vitro data further indicates that genistein also enhances this anti-apoptotic protein expression in endothelial cells. Genistein also decreased TGF-β1, -β2, and -β3 expressions and more importantly the agent also modulated BAX/Bcl-2 ratio [26]. Moreover, the expression of p53 was decreased following genistein treatment as well.

Estradiol also enhanced the expressions of fibroblast growth factor (FGF)-2 and VEGF [2]. Although, combination of these growth factors have been successfully tested to enhance blood reperfusion in affected tissues decreasing the extent of flap necrosis [27], the safety of this approach has remained controversial [28]. An experiment conducted in rabbits revealed that estradiol increases VEGF and NO synthesis and simultaneously decreased malonyldialdehyde production and neutrophil infiltration [29]. Among genes up-regulated in HUVECs, we found pro-inflammatory chemokine CXCL5 and cytokine IL-6 [30,31] as well as pro-angiogenic genes VEGF-A, CTGF, ITGB3, and MMP-14 [32,33,34] following genistein treatment in vitro. On the other hand, genistein also up-regulated the expression of three potent anti-angiogenic molecules, i.e., TIMP-2, -3 (both tumor suppressor genes), and COL18A1 (proteolytic processing at several endogenous cleavage sites in its C-terminal domain results in production of endostatin, a potent anti-angiogenic protein) [35]. However, the expression of these genes was not deregulated in HUVECs in the absence of VEGF. Under this condition, only two genes were down-regulated (CXCL10 and SERPINF1) compared to control. From this point of view, the present data support the tricky effects of SERMs that have the unique ability to selectively act as agonists or antagonists in a tissue-specific manner. Therefore, genistein may improve skin repair [15,36] and simultaneously may exert anti-cancer and chemopreventive properties which has led to several clinical trials combining genistein and/or its analogues with standard of care chemotherapeutic regiments [37,38].

Flavonoids and isoflavonoids have also been shown to influence intracellular redox status, to interact with specific signaling molecules, and to have antioxidant properties [39,40]. In this context, in atherosclerosis, it has been shown that genistein restrains ROS and MDA production, and ameliorates the inhibitory effect on SOD, CAT, glutathione (GSH), and glutathione peroxidase (GPx) activity elicited by ox-LDL stimulation [41]. The effect of genistein was correlated with the up-regulation of sirtuin-1 via inhibiting miR-34a leading to nuclear translocation and deacetylation of foxo3a accompanied with enhanced expressions of MnSOD and CAT. Similarly, in diabetic animals, it was demonstrated dietary genistein supplementation accelerated wound healing by regulation of NF-κB-related inflammatory response (by reducing TNF-α) and Nrf2-associated anti-oxidant defense system [42]. However, in the present study, we found that genistein increases the activity of SOD, but not CAT, in endothelial cells in vitro, using a different (peroxide-induced) oxidative stress model. Of note, we evaluated the SOD/CAT activities only in one cell population (endothelial cells) located in skin and not in other cell types, e.g., skin fibroblasts and/or keratinocytes, which may be the focus of further studies. Furthermore, we also showed that genistein does not modulate ROS production at the most effective wound-healing-promoting concentration of 100 nM. On the other hand, it has also been shown that higher micromolar genistein concentrations exhibit pro-oxidant effects [43]. Therefore, it may be speculated that the protective effect of this phytoestrogen is rather based on the estrogen receptor-mediated anti-apoptotic effect than on its anti-oxidant properties.

## 4. Materials and Methods

### 4.1. Animal Model

The experiment was approved by the Ethical Committee of the Faculty of Medicine of Pavol Jozef Šafárik University in Košice and by the State Veterinary and Food Administration of the Slovakia.

Female Sprague-Dawley rats (*n* = 48), six months of age, were used in this study. These were randomly divided into two basic groups (Table 1). For all surgical interventions we used the same protocol of general anesthesia which was induced by the intramuscular administration of ketamine (40 mg/kg), xylazine (15 mg/kg), tramadol (5 mg/kg), and atropine (0.05 mg/kg).

**Figure 8:**
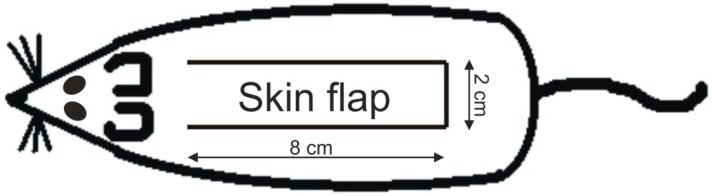
Position of the skin flap. Cranially based skin flap with dimensions 2 × 8 cm placed on the back of a rat.

Three months prior to the flap surgery, rats underwent either ovariectomy (OVX-group) or were sham operated (NOVX-group). Consecutively, under standard aseptic conditions, proximally based over-dimensioned random pattern skin flaps measuring 2 × 8 cm were dissected free from the underlying fascia on the back of each animal (Figure 8). The flaps were immediately returned to their bed and fixed into position by intradermal running suture (Chiraflon 5/0, Chirmax, Prauge, Czech Republic).

All animals were sacrificed seven days after flap surgery and obtained samples were processed for the measurement of necrosis area and histological evaluation.

### 4.2. Genistein Treatment

After flap surgery, rats from all control groups received daily intramuscular injection of saline, while treated animals were administered with 1 mg/kg [36] of genistein (Tocris Bioscience, Bristol, UK). Treatment started either at day of flap surgery or three days prior the flap surgery. The treatment protocol is described in detail in Table 2.

### 4.3. Measurement of Survived Area on the Skin Flap

The flap survival was measured from standardized photographs as follows. Flaps were photographed with a scale immediately after surgery and at days seven using an Olympus E330 digital camera equipped with ED 50 mm f 2.0 macro objective and a ring set flash SRF-11 (Olympus, Tokyo, Japan). The surviving flap area was then measured on the images using Quick PHOTO MICRO 2.2 software (Premiere, Prague, Czech Republic) and expressed as a percentage of the original flap area generated on the day of surgery.

### 4.4. Basic Histology

**Figure 9:**
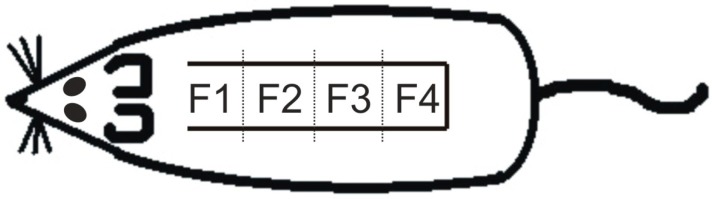
Formaldehyde-fixed skin flaps were cut into four (craniocaudal direction F1, F2, F3, and F4) 2-cm-long specimens that were routinely processed for light microscopy. For better craniocaudal orientation, the flap is shown in its original position on the rat’s dorsum.

All skin flaps were first fixed for 48 h in 4% buffered formaldehyde, then cut into four 2-cm-long specimens (Figure 9) and routinely processed for light microscopy (dehydration, paraffin embedding, sectioning (5 μm), and staining). Hematoxylin-eosine was used for basic staining.

### 4.5. Endothelial Cells

Human umbilical vein endothelial cells (abbreviated as HUVECs) were isolated, cultured, and characterized as previously described [44,45]. Cells were cultured on gelatin-coated dishes in cM199 (M199 medium supplemented with 20% heat-inactivated new born calf serum (both from Cambrex, Verviers, Belgium), 150 μg/mL crude endothelial cell growth factor (ECGF), 5 U/mL heparin, 100 IU/mL penicillin, and 100 μg/mL streptomycin) at 37 °C under 5% CO_2_/95% air atmosphere. Twenty-four hours prior to the experiments, the endothelial cell cultures were refreshed with a serum-reduced (10%) medium without crude endothelial cell growth factor. Cell viability, estimated by trypan blue exclusion, was greater than 95% before each experiment.

Human dermal microvascular endothelial cells (abbreviated as HMVEC-D or HMVECs) were purchased from Lonza (Lonza Walkersville, Inc., Walkersville, MD, USA) and cultured according to the supplier’s instructions. Briefly, cells were cultured in EGMTM-2MV (endothelial growth medium), containing basal medium supplemented with human epidermal growth factor (hEGF), vascular endothelial growth factor (VEGF), R3-insulin like growth factor-1 (R3-IGF-1), ascorbic acid, hydrocortisone, human fibroblast growth factor-beta (hFGF-β), fetal bovine serum (FBS), and gentamicin/amphotericin-β (GA) solution (Lonza, USA) at 37 °C under 5% CO_2_/95% air atmosphere.

### 4.6. Cell Viability Assay

Cell viability of HUVECs were determined using colorimetric microculture assay with MTS (3-(4,5-dimethylthiazol-2-yl)-5-(3-carboxymethoxyphenyl)-2-(4-sulfophenyl)-2H-tetrazolium) dye (Promega, Madison, WI, USA). Cells were seeded at a density of 4 × 10^3^ cells/well in 96-well polystyrene microplates. Twenty-four hours after cell seeding, different concentrations (1–100,000 nM) of the compound in the presence or absence of 25 ng/mL of VEGF were tested. After 48 h of incubation, 10 μL of MTS were added to the each well. After an additional 3 h, cell proliferation was evaluated by measuring the absorbance at wavelength 490 nm using the automated Cytation™ 3 Cell Imaging Multi-Mode Reader (Biotek, Winooski, VT, USA). Absorbance of control wells was taken as 100%, and the results were expressed as a percentage of the untreated control.

### 4.7. In Vitro Migration (Wound Healing) Assay

The motility of HUVECs was assayed using a wound healing assay [46]. Briefly, endothelial cells were cultured on a 24-well plate in the cM199 medium until confluent. A 2 mm pipette tip was used to wound the monolayer of cells. Afterwards, the medium was replaced with fresh serum-reduced medium without ECGF containing the studied compound at different concentrations (1–1000 nM) in the presence or absence of 25 ng/mL VEGF. The wounded area was photographed at the start (*t* = 0 h) and at a specific time point *t* = 16 h. The migration distance (gap size) was determined using image analysis software. The experiments were performed in duplicate wells and repeated three times with cells from different donors.

### 4.8. RNA Isolation and cDNA Synthesis

After treatment of HUVECs with genistein (at final concentration of 100 nM for 48 h) in the presence or absence of 25 ng/mL VEGF, the total cellular RNA was isolated from the cells by a Qiagen RNeasy^®^ Mini Kit (Catalog # 74104) according to the manufacturer’s instructions. RNA samples underwent DNase treatment and removal. RNA quantification was performed with spectrophotometry (ND-1000; NanoDrop Products, Thermo Fisher Scientific, Wilmington, DE, USA), after which 250 ng of total RNA was analyzed by agarose gel electrophoresis to confirm integrity. The resultant RNA was stored at −80 °C. Only samples pure enough (A260/A230 ratio > 1.8, A260/A280 ratio = 1.8–2.0), with reasonable concentration (>100 ng/μL), were used as templates for cDNA synthesis. First-strand complementary DNA was synthesized from total RNA (0.5 μg) using the RT2 First-Strand Kit (Catalog # 330401, Qiagen, Germany). In brief, 0.5 μg of total RNA was added to 2 μL of Buffer GE (5× gDNA Elimination Buffer), and the final volume was made up to 10 μL with RNase-free water. The mixture was denatured at 42 °C for 5 min and then immediately cooled by placing on ice for 1 min. Reverse transcription was performed after adding 10 μL of reverse transcription mix to the solution. The reaction mixture was incubated at 42 °C for 15 min, after which it was terminated by heating at 95 °C for 5 min. The cDNA samples generated were then diluted with 91 μL RNase-free water and stored at −20 °C until further analysis.

### 4.9. Gene Expression Profiling

Real-time PCR was carried out by using a Biorad CFX96. Gene expression was examined using the Human Wound Healing RT2 Profiler™ PCR Array (cat # 330231D, Qiagen, Germany). The RT2 Profiler™ PCR Array contains built-in primers for 84 tested and 5 housekeeping genes and positive control elements to determine the efficiency of the reverse transcription reaction, performance of the PCR reaction, and detection of genomic DNA contamination. The PCR mixture for 96 reactions contained 1350 μL of RT2 SYBR Green Mastermix (Qiagen, Germany), 102 μL cDNA template, and 1248 μL RNase-free water. The PCR reaction mix was added to the wells of the PCR plate in equal amounts (25 μL), and then the real-time PCR cycling program was run. The thermal cycling program recommended by plates manufacturer for Biorad CFX96 was as follows: 10 min at 95 °C followed by 40 cycles: denaturation at 95 °C for 15 s, with 60 s annealing and elongation at 60 °C, followed by melting curve analysis.

### 4.10. PCR Data Analysis and Statistics

RT profiler data were analyzed using SABiosciences data analysis software (http://pcrdataanalysis.sabiosciences.com/pcr/arrayanalysis.php). The ΔΔCt method was used for data analysis. Specifically, fold-changes for each gene were calculated as difference in gene expression between genistein exposure in the presence or absence of growth factor and control. A positive value indicated gene up-regulation and a negative value indicated gene down-regulation. Each experiment was independently repeated at least twice (as recommended by the manufacturers guidelines for statistical significance). Genes with greater than 2.0-fold change in expression compared to control were identified as significant (*p* < 0.05).

### 4.11. Flow Cytometric (FCM) Analysis of Genistein Effect on Pro-/Anti-Apoptotic Gene Expressions

For FCM analysis, floating and adherent cells were harvested together 48 after treatment. After centrifugation, the cells were washed in PBS, divided for particular analysis, and stained prior to analysis (see Table 4). Fluorescence was detected after 15–30 min incubation in the dark at room temperature using a FACSCalibur flow cytometer (Becton Dickinson, San Jose, CA, USA). A minimum of 1 × 10^4^ events were analyzed per analysis.

### 4.12. CAT and SOD Activity

HMVEC-D and HUVEC cells were seeded in 6-well plates in duplicates (1,000,000 cell/2 mL) and pre-incubated with genistein (Tocris) (10, 100, 1000 nM) for 72 h. Medium without genistein was used as control. Following pre-incubation, medium was changed to medium supplemented with genistein (Tocris) (10, 100, 1000 nM) and/or H_2_O (Sigma-Aldrich, St. Louis, MO, USA) (0.5 mM) was added to the cells and incubated for 5 h. The activities of CAT and SOD were determined using assay kits according to the manufacturer instructions (Cayman Chemical Company, Ann Arbor, MI, USA).

### 4.13. Detection of Intracellular Oxidative Stress

The generation of reactive oxygen species (ROS) was assessed by using a DCFH-DA probe. The HMVEC-D cells were seeded in the black plate 48,000 cells/100 μL/well in standard culture medium. After 24 h, the medium was replaced with serum free medium. After 1 h incubation, genistein was added in three concentrations (10, 100, and 1000 nM), 45 min later DCFH-DA (10 μM) was added and after 15 min hydrogen peroxide (0.5 mM) was added. Following 60 min, the intracellular fluorescence of DCF was measured (λ (excitation/emission) = 480/530) using an Infinite M200 spectrofluorometer (Tecan, Männedorf, Switzerland) and compared to the blank. Genistein and DCFH-DA were diluted in DMSO (not exceed 0.5% at final concentration in culture medium).

### 4.14. Statistical Analysis

Two-way analysis of variance (ANOVA) followed by Tukey–Kramer test were used to compare the differences in percentages of flap viability areas (that are presented as mean ± SD). CAT/SOD activities and presence of ROS were evaluated using one-way ANOVA followed by Tukey–Kramer test. Significance was accepted at *p* < 0.05.

## 5. Conclusions

In conclusion, in the present study we have demonstrated that genistein exert significant tissue ischemia protective effects only when applied three days prior the flap surgery. Moreover, based on our in vitro study, the basic molecular mechanism may be related to the up-regulation of Bcl-2 expression and SOD activity. Since genistein has never been previously tested in an ovariectomized model of skin flap, our current results provide new information to previously observed effects of estrogens and correlates to previously published studies where it has been shown that post-surgical estradiol/genistein treatment significantly improves wound healing. Of note, the use of an ER-α and ER-β deficient animal model or administration of genistein in combination with a highly selective ER antagonist would bring more light into the explanation of its signaling via ER-pathways.

Here, we presented the positive effect of genistein on skin flap viability. Several on-going clinical trials point to the safety and efficiency of this drug for the use in cancer prevention/treatment [47,48] and regenerative medicine [49]. Nevertheless, further research, including the discover of the exact underlying mechanisms and optimal treatment protocol, needs to be performed so that these findings may be applied in clinical practice.

## Figures and Tables

**Table 1:** Animals and groups. Animals were assigned to 6 groups based on the surgical procedure (ovariectomy vs. sham surgery) and administered treatment protocol.

Group/Subgroup		No. of Rats	Ovariectomy (O)/ Sham Surgery (S)	Treatment Started	Dose mg/kg
NOVX	-C	8	S—3M prior flap surgery	from the day of flap surgery	0
	-G0	8	S—3M prior flap surgery	from the day of flap surgery	1
	-G3	8	S—3M prior flap surgery	3 days prior flap surgery	1
OVX	-C	8	O—3M prior flap surgery	from the day of flap surgery	0
	-G0	8	O—3M prior flap surgery	from the day of flap surgery	1
	-G3	8	O—3M prior flap surgery	3 days prior flap surgery	1

**Table 2:** Fold change of gene expression in HUVECs exposed to genistein (100 nM) in the presence of VEGF compared to VEGF-treated control.

Gene Symbol	Gene Name	Genistein + VEGF/VEGF
COL18A1	Collagen Type XVIII Alpha 1 Chain	2.2
CTGF	Connective Tissue Growth Factor	2.4
CXCL5	C-X-C Motif Chemokine Ligand 5	2.8
IL-6	Interleukin 6	2.1
ITGB3	Integrin Subunit Beta 3	2.6
MMP-14	Matrix Metallopeptidase 14	2.4
TIMP-2	TIMP Metallopeptidase Inhibitor 2	2.2
TIMP-3	TIMP Metallopeptidase Inhibitor 3	2.2
VEGF-A	Vascular Endothelial Growth Factor A	2.4

**Table 3:** Fold change of gene expression in HUVECs exposed to genistein (100 nM) compared to untreated control.

Gene Symbol	Gene Name	Genistein/Control
CXCL10	C-X-C Motif Chemokine Ligand 10	−2.7
SERPINF1	Serpin Family F Member 1	−2.1

**Table 4:** Reagents used for FCM analysis of endothelial cells following genistein treatment.

Analysis	Staining Solution	Manufacturer
Protein analysis	Bcl-2 (124) Mouse mAb (PE Conjugate) 1:50	Cell Signaling Technology, Danvers, MA, USA
	Phospho-Bcl-2 (Ser70) Rabbit mAb (Alexa Fluor^®^ 488 Conjugate) 1:50	
